# Self-supervised learning mechanism for identification of eyelid malignant melanoma in pathologic slides with limited annotation

**DOI:** 10.3389/fmed.2022.976467

**Published:** 2022-09-27

**Authors:** Linyan Wang, Zijing Jiang, An Shao, Zhengyun Liu, Renshu Gu, Ruiquan Ge, Gangyong Jia, Yaqi Wang, Juan Ye

**Affiliations:** ^1^Department of Ophthalmology, The Second Affiliated Hospital, Zhejiang University School of Medicine, Hangzhou, China; ^2^School of Computer Science and Technology, Hangzhou Dianzi University, Hangzhou, China; ^3^Department of Pathology, Lishui Municipal Central Hospital, Lishui, China; ^4^College of Media Engineering, The Communication University of Zhejiang, Hangzhou, China

**Keywords:** artificial intelligence - assisted bioinformatic analysis, self-supervised deep learning, pathology, tumor diagnosis, melanoma

## Abstract

**Purpose:**

The lack of finely annotated pathologic data has limited the application of deep learning systems (DLS) to the automated interpretation of pathologic slides. Therefore, this study develops a robust self-supervised learning (SSL) pathology diagnostic system to automatically detect malignant melanoma (MM) in the eyelid with limited annotation.

**Design:**

Development of a self-supervised diagnosis pipeline based on a public dataset, then refined and tested on a private, real-world clinical dataset.

**Subjects:**

A. Patchcamelyon (PCam)-a publicly accessible dataset for the classification task of patch-level histopathologic images. B. The Second Affiliated Hospital, Zhejiang University School of Medicine (ZJU-2) dataset – 524,307 patches (small sections cut from pathologic slide images) from 192 H&E-stained whole-slide-images (WSIs); only 72 WSIs were labeled by pathologists.

**Methods:**

Patchcamelyon was used to select a convolutional neural network (CNN) as the backbone for our SSL-based model. This model was further developed in the ZJU-2 dataset for patch-level classification with both labeled and unlabeled images to test its diagnosis ability. Then the algorithm retrieved information based on patch-level prediction to generate WSI-level classification results using random forest. A heatmap was computed for visualizing the decision-making process.

**Main outcome measure(s):**

The area under the receiver operating characteristic curve (AUC), accuracy, sensitivity, and specificity were used to evaluate the performance of the algorithm in identifying MM.

**Results:**

ResNet50 was selected as the backbone of the SSL-based model using the PCam dataset. This algorithm then achieved an AUC of 0.981 with an accuracy, sensitivity, and specificity of 90.9, 85.2, and 96.3% for the patch-level classification of the ZJU-2 dataset. For WSI-level diagnosis, the AUC, accuracy, sensitivity, and specificity were 0.974, 93.8%, 75.0%, and 100%, separately. For every WSI, a heatmap was generated based on the malignancy probability.

**Conclusion:**

Our diagnostic system, which is based on SSL and trained with a dataset of limited annotation, can automatically identify MM in pathologic slides and highlight MM areas in WSIs by a probabilistic heatmap. In addition, this labor-saving and cost-efficient model has the potential to be refined to help diagnose other ophthalmic and non-ophthalmic malignancies.

## Introduction

Malignant melanoma (MM) is an intractable cutaneous cancer originating from melanocytes with an extremely high mortality rate (65% of all skin cancer deaths) (1). Although eyelid melanoma accounts for only ∼1% of all cutaneous melanomas, it can camouflage melanocytic nevus (the most common benign eyelid tumor) both in the naked eye and under a microscope. Its primary diagnosis and management fall within the realm of ophthalmology. Despite the similar appearance, these two tumor types have markedly different biological behaviors, corresponding to distinct prognoses and treatments. Therefore, it is critically important to distinguish between the two diseases (2, 3). Like other types of tumor, the gold standard for MM diagnosis still relies on manual histopathological interpretation, which is subjective, laborious, tedious, and challenging for pathologists and ophthalmologists lacking experience encountering eyelid melanoma (3). Computer-aided diagnosis (CAD) in eyelid melanoma cases is urgently needed to make a comprehensive and objective pathological diagnosis (4).

The advancement of artificial intelligence (AI) technology has cast light both on natural images and medical areas. Compared with other fields, the automatic diagnosis based on histopathological images confronts more challenges due to the uniqueness of pathological data. Firstly, the digitization of traditional glass slides needs additional scanning equipment. Secondly, most pathological images are gigapixels, which are tremendously large: about 470 whole slide images (WSIs) scanned at 20× magnification (0.5 μm pixel^–1^) contain roughly the same number of pixels as the entire ImageNet dataset (5). The diagnosis of pathology highly depends on its cellular characteristics, which means we need to annotate and analyze at the patch (a small tile cutting from WSI) level first. Such a procedure requires tremendous annotations by expert pathologists. Thirdly, based on the incidence of ocular tumors, the pathology slides are more valuable than fundus images or optical coherence tomography (OCT) images, which could be obtained in a routine follow-up. The lack of expertise to make high-quality annotations further restricted the number of usable pathology slides.

However, the availability of medical specialists to annotate digitized images and free text diagnostic reports does not scale with the need for large datasets required to train robust computer-aided diagnosis methods that can target the high variability of clinical cases and data produced. Most previous attempts in computational pathology are fully supervised learning studies. The automated system of pathological images requires a sufficient quantity of images with annotations (6–9). There are several drawbacks to this procedure. First, collecting unlabeled digitized slides only needs technicians to scan, but labeled images need extra experts with many years of medical education. If unlabeled medical images could be used in deep learning analysis, the usable datasets could be significantly expanded. Moreover, the laborious annotation has the potential to introduce manual label errors, as most current annotations were carried out at lower magnification.

Moreover, some boundaries of the tumor area are ambiguous with normal mixed cells and cancer cells, which even perplexes the annotation process. Last but not least, the unlabeled images by themselves may still include substantial clinical information. From the view above, generating a diagnostic system that can utilize labeled and unlabeled images may greatly benefit the diagnosis and treatment of the disease.

Self-supervised learning (SSL) is a new type of unsupervised learning algorithm to extract and analyze features of given data automatically. SSL has been applied to input data in various models, including RGB image (10), videos (11), medical image (12), mass spectrometry data (13), or multimodal data (14). The high efficiency of SSL makes it suitable for auxiliary medical uses. SSL requires only a limited quantity of labeled data and a relatively abundant quantity of unlabeled data for the machine to learn features. This perfectly meets the clinical conditions in which annotating pathological images is laborious, time-consuming, and probably inaccurate. We generate a diagnostic system based on Bootstrap Your Own Latent (BYOL), a new approach to SSL proven to achieve better performance compared to contrastive methods of other SSL algorithms (15). Generally, an original gigapixel-level pathological image is too complex for a deep learning system (DLS) to analyze. Therefore, we divided images into small patches. After pretreating these patch-level images, we input the patches of unlabeled images into the SSL network for extracting features as a pretraining task. Subsequently, combined with other labeled images, these learned features are repurposed to improve the classification of the network and thus increase data utilization. Using a random forest model, we extrapolated patch-level classification to whole-slide-image-level classification. Apart from the above, we also generate a heatmap of pathologic images to interpret the decision-making process.

This study aims to apply an SSL network to diagnose and classify MM and non-malignant areas from digital H&E stained pathological slides. To our knowledge, no other SSL networks have been used in detecting eyelid melanoma; we demonstrate the feasibility of using limited labeled data to establish a reliable eyelid MM detection model and describe a strategy for highlighting specific areas of concern.

## Methods

**FIGURE 1:**
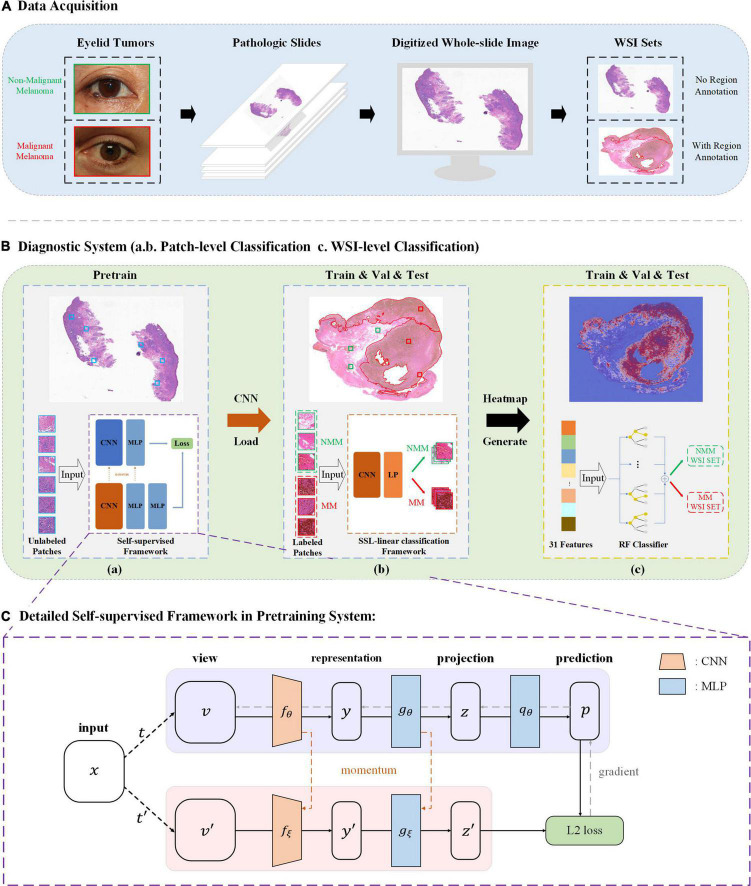
Study workflow. **(A)** Pathologic slides were acquired from eyelid tumors and transformed into digitized whole-slide images (WSIs). An experienced pathologist labeled ∼25% WSIs by delineating the tumor areas in WSIs. **(B)** Diagnostic system. **(a)** Pretraining is based on Bootstrap Your Own Latent (BYOL), a new approach to SSL. Patches from unlabeled WSIs were input into two identical convolutional neural networks (CNNs) with two different sets of weights for learning features and comparing the outputs with each other as pretraining. A load of learned image representation was then generated. **(b)** Training for patch-level classification. Patches from labeled WSIs (training and validation sets) were input into a CNN for training together with the load from the pretraining round, and training weights were acquired. The diagnostic ability of patch-level images was evaluated in the testing set. A value of the malignancy probability of every patch is then generated (not shown). **(c)** Extrapolation to image-level classification. Patches were embedded back into the corresponding WSIs, and by feeding back the malignancy probability of every patch, a probabilistic heatmap for WSIs was generated. Based on the predicted patch value, the threshold transformation was used to extract 31 features. The WSI-level classification based on random forest (RF) was then assigned. **(C)** BYOL architecture. In 2 CNNs (*f*θ and *fξ*) with a different set of weights, θ are the trained weights, and *ξ* is an exponential moving average of θ. At the end of the training, parameter θ is acquired with the minimum of L2 loss, and *y* is used as the learned representation—Val, validation; MLP, multilayer perceptron; MM, malignant melanoma; NMM, non-malignant melanoma.

This study was approved by the Second Affiliated Hospital, Zhejiang University School of Medicine (ZJU-2) Ethics Committee (No. Y2019-195) and the study adhered to the Declaration of Helsinki. In this study, we applied self-supervised learning to make eyelid melanoma identification. Our algorithm was first developed and tested in PatchCamelyon and then in the ZJU-2 dataset. Digitized pathologic images of slides were cut into small patches. The classification was based on these patch-level images and then extrapolated to WSIs. Besides, the algorithm also generated a heatmap to highlight the exact lesion area in WSI and improve the interpretability of the decision-making process of our model. The whole study workflow is summarized in Figure 1.

### Datasets

**FIGURE 2:**
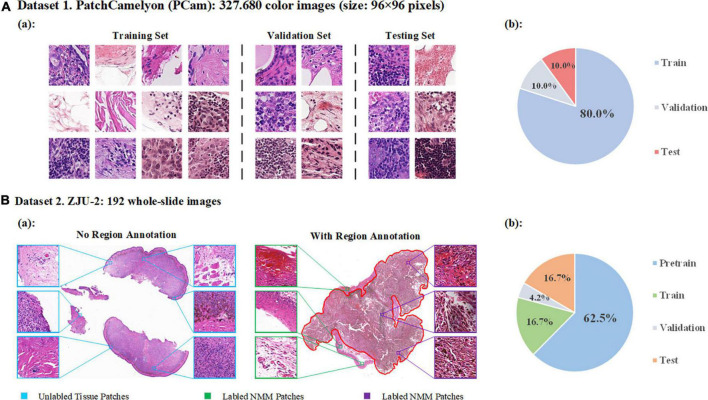
Data distribution. **(A)** Detailed data of PatchCamelyon (Pcam). **(a)** Examples of images in PatchCamelyon. **(b)** The data of original image grouping. **(B)** Dataset of ZJU-2. **(a)** Examples of pathological digitized WSIs with or without annotations and patches from WSIs. **(b)** Patches are divided into four sets: pretraining, training, validation, and testing sets.

A. PatchCamelyon (PCam): a publicly accessible dataset containing 327680 annotated color images (96 × 96 pixels) extracted from histopathologic scans of lymph node sections (16). The dataset uses agreed-upon metrics widely to compare different convolutional neural networks (CNNs) as the backbone. In this study, we used PCam as the benchmark for our model and compared the performance of our model to other CNNs or algorithms. The original data of PCam is shown in Figure 2A. The images were divided into training, validation, and testing.

B. ZJU-2 dataset: 192 whole-slide images (WSIs) from formalin-fixed paraffin-embedded (FFPE) pathological slices (Table 1). We retrospectively included 160 patients from the Second Affiliated Hospital, Zhejiang University School of Medicine, between January 2005 and December 2017, without other types of special selection. All slides were diagnosed by a minimum of two board-certified pathologists using H&E staining (if necessary, additional immunohistochemical staining was used) and traditional microscopy. There was no divergence in the diagnosis of all samples in this study. A separate technician working within the pathology department then scanned the selected slides and digitized these slides into WSIs at 400-fold magnification using a KF-PRO-005 (KFBio, Zhejiang, China). The WSIs were divided into four sets: pretraining, training, validation, and testing set. Besides the WSIs in the pretraining set, images from the other three sets were reviewed and labeled by an additional independent pathologist (>10 years of experience). By using window sliding, 192 whole slide images (WSIs) were cut into a total of 524,307 patches (256 × 256 pixels) for analysis (Figure 2B). The detailed image data partition is shown in Table 1. It’s worth noting that only delineated tumor areas of MM slides were defined as malignant, and other patches were defined as non-malignant.

**TABLE 1:** Summary of the ZJU-2 dataset.

Data set	Pretraining set		Training set		Validation set		Testing set		Total set	
	(Unlabeled)		(Labeled)		(Labeled)		(Labeled)			
Images (n)	WSI (patient)	Patch	WSI (patient)	Patch	WSI (patient)	Patch	WSI (patient)	Patch	WSI (patient)	Patch
Non-malignant	80 (76)		24 (24)	34187	4 (4)	1099	24 (24)	15597	132 (123)	
Malignant	40 (24)		8 (8)	35620	4 (4)	874	8 (8)	14762	60 (37)	
Total	120 (100)	422168	32 (32)	69807	8 (8)	1973	32 (32)	30359	192 (160)	524307

### Self-supervised learning approach

We used Bootstrap Your Own Latent (BYOL) for learning features from unlabeled WSIs in this study (15). The study workflow was shown in Figures 1A–C. The architecture is shown in Figure 1C. In detail, it consists of two neural networks: online networks and target networks. It produces two augmented views (*v* and *v’*) from a single image by applying two different distributions of image augmentations: one with a random horizontal flip and another with a random horizontal flip and gaussian blur (*t* and *t’*). Two identical CNNs with a different set of weights then output the representation (*y* and *y’*) and projection (*z* and *z’*) through a multilayer perceptron (MLP). On the online branch, we output the prediction *p*, making the architectural asymmetry. After normalizing *p* and *z’*, we defined the mean squared error between normalized predictions and target projections, thereby generating the loss (${ℒ}_{\theta ,\xi }^{\mathrm{SSL}-\mathrm{linear}}$). By reversely feeding *t* to the target network and *t’* to an online network, we computed the loss (${ℒ}_{\theta ,\xi^{\prime }}^{\mathrm{SSL}-\mathrm{linear}}$) and minimized the L2 loss = ${ℒ}_{\theta ,\xi }^{\mathrm{SSL}-\mathrm{linear}}+{ℒ}_{\theta ,\xi^{\prime }}^{\mathrm{SSL}-\mathrm{linear}}$ with a stochastic optimization step, as depicted by the unidirectional gradient in Figure 1C. The Adam optimizer is used, and the learning rate is set as 3e-4. The learning rate of SSL-linear is 0.01, and the momentum is set at 0.9. Four NVIDIA TITAN Xp GPUs were used for model training.

### Self-supervised learning-linear for patch-level classification

The self-supervised algorithm needs to use a CNN model as its base algorithm or backbone. When choosing the backbone candidates, we took the size of our datasets and the depth, stability, and memory cost of different CNNs into consideration when choosing the backbone candidates. So, we started with five commonly used CNNs (VGG16, ResNet18, ResNet50, DenseNet121, EfficientNetB7) for initial fully supervised learning tests in PCam (17, 18). After choosing the CNN with the best performance as the backbone to generate the SSL-linear, we moved on to the next experiment stage–comparing different self-supervised algorithms. The SSL-linear (No Pre) and SSL-linear (Frozen) methods were used as control groups to prove that both stages are necessary for the SSL-linear method. SSL-linear (No Pre) did not undergo a pretraining process, and SSL-linear (Frozen) froze the backbone CNN model’s parameters during the training process, which is a traditional way of self-supervised learning. By comparing SSL-linear to the traditional classifiers, including ResNet50, SSL-linear was proved to be valid and feasible for pathological images. The algorithm was then applied to learn features and make classifications from patch-level images in four sets of the ZJU2 dataset.

Model performance was evaluated by accuracy (Acc), sensitivity (Sen), specificity (Spe), and the κ statistic (Cohen’s kappa coefficient). For every patch, malignancy probability was calculated between 0 and 1 (1 refers to definitively malignant and is presented in red on the heatmap, while 0 refers to completely NM and is presented in blue) before feeding this estimate back into the WSI and generating the probabilistic heatmap for the full WSI.

### Feature extraction and whole-slide-image-level classification using random forest

The original probabilistic heatmap was reprocessed, and 31 features were extracted, including the number of tumor areas; the proportion of tumor areas in the whole tissue; the largest area of the tumor; the longest axis of the largest tumor area; the prediction value across the tumor areas; the number of positive pixels; max, mean, variance, skewness, and kurtosis of pixel numbers in all tumor areas; perimeter, eccentricity, and solidity in tumor areas (6). These features were then used for WSI-level classification. The probabilistic input heatmap was a single-channel image the same size as the original WSI. Each pixel was refilled based on the prediction results (malignancy probability between 0 and 1). The 31 tumor features were encompassed with a threshold of 0.5. For all input objects, pixels greater or equal to the threshold value were assigned a pixel value of 255, while those below the threshold were set to 0. Following the extraction of these 31 features, WSI-level classification was applied. The random forest classifier shared the same training sets as SSL-linear, but SSL-linear analyzed patch-level images while the random forest classifier analyzed WSIs. The extracted 31 features with label information were sent into the random forest model for prediction.

### Statistical analysis

In this study, we plotted receiver operating characteristic (ROC) curves to evaluate the performance of different classification algorithms. Classification metrics were calculated, including Acc, Sen, Spe, κ score (Cohen’s kappa), balanced accuracy (B_Acc), and the area under the receiver operating characteristic curve (AUC) for each model. B_Acc is more sensitive to imbalanced data and can be used to address the inequality between malignant and NM data sets. All statistical analyses were conducted using the programming language Python (V.3.5.4).

## Results

### Classification ability in PatchCamelyon – the public dataset

In the PCam dataset, ResNet 50 outperformed the other four commonly used CNNs (VGG16, ResNet18, DenseNet121, and EfficientNetB7) in the supervised study task (Table 2, Group 1) and was chosen as the backbone to generate the SSL-linear algorithm. In the supervised study, among these 5 CNNs, ResNet50 had the highest AUC, 0.950, spe 90.1%, indicating the best performance. EfficientNetB7 had the highest Acc 88.4%; B_Acc 88.4%; κ score 0.767; Spe 92.0%. However, the training time of EfficientNetB7 (83.6 h) is approximately 5.5 times longer than ResNet50 (14.7 h). The volume of parameters of EfficientNetB7 (63.8 M) is 2.7 times larger than ResNet50 (23.5 M). The long training time and high demand for the memory capacity of graphical processing units (GPUs) make it impractical to use EfficientNetB7 in clinical settings. The comparison experiments of various networks also verified the rationality of the selection. Second, in a self-supervised study, we evaluated and compared the performance of SSL-linear and ResNet50 with different proportions of unlabeled pretraining and labeled training sets (Table 2, Group 2 and Group 3). Notably, the pretraining set was derived from the original training set in PCam. The single ResNet50 could not perform self-supervised learning from the pretraining set, so when we compared ResNet50 with SSL algorithms, ResNet50 only learned features from the training set, which was identical to patches in the training sets of other SSL algorithms. The results presented that in both the 5:5 and 7:3 proportions we used in this task, SSL linear achieved the best overall performance. The AUC, Acc, B_Acc, κ score were 0.939, 86.1%, 86.1%, 0.723 for the 5:5 proportion and 0.932, 85.4%, 85.4%, 0.709 for the 7:3 proportion, which were higher than other groups. It was worth noting that AUC, Acc, B_Acc, κ score, and spe of SSL-linear in the 7:3 proportion group were higher than ResNet50 in the 5:5 proportion group, indicating that SSL-linear utilized less labeled patches but achieved a better performance than ResNet50. Although the performance of SSL-linear didn’t exceed that of the other four state-of-the-art supervised learning algorithms, SSL-linear utilized only half or even less labeled data to achieve accuracy with a gap smaller than 5%. The results proved that SSL-Linear was both valid and feasible for patch-level classification in pathological slide images, even with a limited amount of labeled data. Detailed information is reported in Table 2.

**TABLE 2:** Results of classification task in Patchcamelyon (PCam).

Pretrain: Train	Method	Acc (%)	B_Acc (%)	κ score	Sen (%)	Spe (%)	AUC
**Ratio**							
0:10 (Group 1)	VGG16	87.8	87.8	0.755	88	87.5	0.949
	ResNet18	85.9	85.9	0.718	82	90	0.929
	ResNet50	88.2	88.2	0.765	**86.3**	90.1	**0.950**
	DenseNet121	87.8	87.8	0.756	85.4	90.2	0.947
	EfficientNetB7	**88.4**	**88.4**	**0.767**	84.7	**92.0**	0.941
	Veeling et al. (17)	89.8					
	Mohamed et al. (18)	89.2					
5:5 (Group 2)	ResNet50 (No Pre)	84.2	84.2	0.685	**84.1**	84.4	0.923
	SSL-linear (No Pre)	84.8	84.8	0.697	78.4	91.2	0.925
	SSL-linear (Frozen)	75.7	75.7	0.514	81.7	69.7	0.828
	SSL-linear	**86.1**	**86.1**	**0.723**	82.3	**89.9**	**0.939**
7:3 (Group 3)	ResNet50 (No Pre)	83.2	83.2	0.664	75	91.3	0.921
	SSL-linear (No Pre)	83.8	83.8	0.677	75.4	**92.3**	0.931
	SSL-linear (Frozen)	74.6	74.6	0.493	**82.8**	66.5	0.82
	SSL-linear	**85.4**	**85.4**	**0.709**	82.6	88.3	**0.932**

The bold term represents the highest score within the same group.

### Patch-level classification of ZJU-2

**FIGURE 3:**
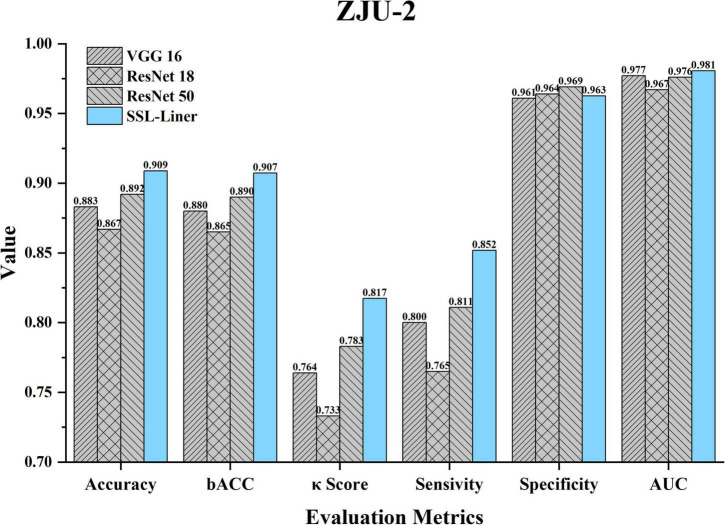
Comparison of different metrics for SSL-linear and 5 CNNs at the patch-level testing set of ZJU-2. κ, unweighted Cohen’s kappa; Acc, accuracy; AUC, area under the receiver operating characteristic curve; B_Acc, balanced accuracy; CNN, convolutional neural network; ZJU-2, The Second Affiliated Hospital, Zhejiang University School of Medicine.

The dataset distribution is summarized in Table 1. The whole set contained 422,168 patches from 120 unlabeled images and 102139 patches from 72 labeled images. The Acc, B_Acc, κ score, Spe, Sen, and AUC were calculated to evaluate and compare SSL-linear and five CNNs (Figure 3). After pretraining, SSL-linear achieved the best performance compared to five CNNS with identical training set, indicating the positive effect of the pretraining round. The Acc, B_Acc, κ score, Sen, and AUC were 90.9%, 90.7%, 0.817%, 85.2%, and 0.981 for SSL-linear, higher than other groups. Detailed information is reported in Figure 3.

### Whole-slide-image-level classification of ZJU-2

**FIGURE 4:**
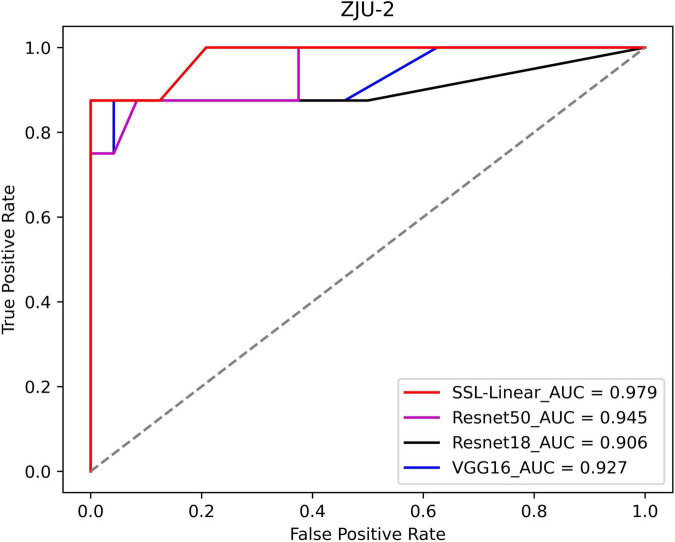
The receiver operating characteristic (ROC) curves of SSL-linear and 5 CNNs. Performance of SSL-linear, VGG16, ResNet18, and ResNet50 for melanoma detection for WSIs from ZJU-2. AUC, the area under the receiver operating characteristic curve; ZJU-2, The Second Affiliated Hospital, Zhejiang University School of Medicine.

In a real-world clinical setting, clinicians worry about the diagnosis of a certain slide instead of the small patches. Thus, we evaluated the WSI classification ability of our algorithm and compared it to five CNNs (Figure 4). The ROC curve was plotted, and AUC was calculated. The AUCs for SSL-linear, VGG16, ResNet18, and ResNet50 were 0.964, 0.935, 0.891, and 0.938, indicating that SSL-linear achieved the best performance in the WSI-level classification task. For 32 WSIs in the testing set of ZJU-2, SSL-linear failed to diagnose two malignant cases. Other metrics were calculated: Spe 100%; Sen 75%; Acc 93.8%; and κ score 0.818.

### Visualization heatmap

**FIGURE 5:**
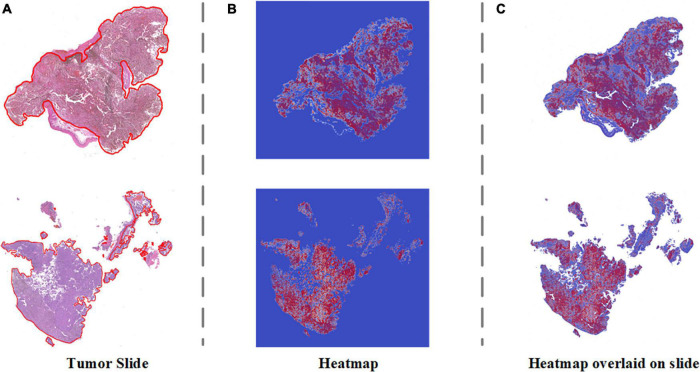
Visualization heatmap of pathological slides based on SSL. **(A)** The original pathological slide with tumor area delineated (H&E staining, ×40 scanned). **(B)** Probabilistic heatmap of the tumor slides generated by the algorithm. Red indicates higher malignancy. **(C)** Overlap of the tumor slide image and probabilistic heatmap.

To address the clinical scenario and increase the interpretability of the diagnosis results of our algorithm, we generated a probabilistic heatmap by integrating the corresponding patches. The melanoma area in the slides was highlighted red and indicated whether the surgical margin was negative. Figure 5 demonstrates how our algorithm suggests melanoma areas by heightening the malignant zone. Figures 5A–C represent the original tumor slide image, the corresponding probabilistic heatmap and the overlap image, respectively. The overlapping image indicates that the prediction area of our algorithm corresponds to the delineation area.

## Discussion

In this study, we trained a self-supervised learning model with a limited number of labeled images and developed a diagnostic system to detect eyelid MM in pathological slides. By comparing the classification ability of VGG16 ResNet18 and Resnet50 in PCam, ResNet50 was selected as the backbone for our pathologic diagnosis algorithm to generate SSL-linear. SSL-linear is based on BYOL and requires two identical CNNs (ResNet50 in this study) with a different set of weights in the pretraining round. In the patch-level classification task, SSL-linear displayed higher diagnostic accuracy even with fewer labeled images than the traditional ResNet50 classifier. We also introduced two state-of-the-art fully supervised algorithms (17, 18) to compare the performance of PCam. While the algorithms are closed source and utilize a training set 2 or 3 times larger than SSL-linear, the performance gap in Acc is relatively acceptable (< 5%). It is valid and feasible for SSL-linear to make patch-level classifications in pathological slides. When applying to the ZJU-2 dataset, SSL-linear also demonstrated high diagnostic ability with the approximate 4:1 proportion of pretraining (unlabeled) and training (labeled) set in the patch-level and gigapixel WSI-level classification tasks. The computing systems that are used to solve problems in AI are opaque (19). This makes the diagnosis provided by the algorithm hard to convince both doctors and patients. To address this issue, we engineered our system to design a probabilistic heatmap highlighting malignant areas for pathologists. The emphasis on the area merits extra attention, and the indication of the negative margin is especially meaningful in highly lethal cancers like melanoma in our case. Recently, there has been constant progress in self-supervised learning methods, such as contrastive learning, clustering, Simple Siamese networks, BYOL, etc., optimizing the performance of SSL algorithms and allowing transfer learning for different tasks (15, 20–22). In the medical field, SSL has been used to solve different problems with the type of input data (12, 13, 23, 24). Among these studies, pathological images have uniqueness for the following reasons. First, the pathology department encounters a huge quantity of slides, most of which won’t be scanned to transfer into WSIs. Second, WSIs are at a gigapixel level with enormous information. Therefore, making annotations of pathologic slides is laborious, time-consuming, and requires a strong medical background. Although some slides were transferred into WSIs, most WSIs were not labeled; third, histopathological interpretation remains the gold standard for diagnosing some diseases. Currently, most research groups focus on improving the accuracy in the field of automatic pathological diagnosis, and different kinds of pathological images have been utilized. For instance, Ström et al. used labeled biopsy for algorithms to diagnose and grade prostate cancer (24); Kather et al. used deep learning to predict whether patients with gastrointestinal cancer respond well to immunotherapy (25).

Despite various motivations, most studies relied on sufficient ground truth labels of WSIs, which is difficult to attain in clinical scenarios. SSL does not require as many labeled WSIs as fully supervised learning and thus demonstrates the natural advantages of dealing with WSI’s diagnosis. Some algorithms based on SSL have been applied to pathology. For example, Wataru et al. used their SSL-based algorithm to predict the pathological diagnosis of patients with interstitial lung disease. The algorithm achieved an AUC of 0.90 in the validation set and 0.86 in the testing set in diagnosing usual interstitial pneumonia with an approximate 1:2 proportion of pretraining and training set (4:1 proportion of pretraining and training set in the ZJU-2 dataset) (26, 27). However, due to the difference in task and labeling strategies, we cannot directly compare the performance of our algorithm to other studies.

Moreover, most studies in this field have focused on predicting common diseases. However, compared with common diseases, which are more unlikely for pathologists to misdiagnose, eyelid MM, the less common and dangerous cancer, presents a more urgent need for automated diagnosis or auxiliary diagnosis due to the lack of experience in encountering MM. Besides, our algorithm SSL-linear makes good use of unlabeled WSIs in reducing the burden of annotation and enhancing data utilization while achieving considerable performance in diagnosis.

To the best of our knowledge, it is the first study to apply self-supervised learning algorithms to ocular pathological research. With the approximate 4:1 proportion of pretraining and training set, SSL-linear achieved high accuracy at detecting MM area both in a patch (98.1%) and at WSI level (93.8%), out-competing the other five traditional CNNs. SSL-linear shows considerable diagnostic ability with limited labeled input data, not only easing the burden of annotating many gigapixel images but also providing relatively reliable diagnostic support for pathologists, especially those less experienced. Additionally, our diagnostic system takes only minutes to generate the output prediction results together with a clear probabilistic heatmap. For patients with MM, our diagnostic system can potentially reduce the probability of misdiagnosis and diagnostic omission, thus promoting the early treatment of MM. For clinicians, they could take advantage of telemedicine for rapid intraoperative consultation feedback. For pathologists, the highly malignant area indicated by the heatmap is also helpful in writing the pathological report and confirming the diagnosis.

Furthermore, it could raise the doctors’ awareness of eyelid MM, a relatively less common cancer with a high mortality rate, and prioritize samples with higher malignant potential for senior pathologists. Despite the advantages of the automatic diagnostic system, human pathologists’ work is still irreplaceable and has its own superiority. In a real clinical setting, the challenging cases will be reviewed by multiple pathologists with the help of immunohistochemistry, molecular information, or even genetic information in addition to H&E staining, while the algorithms only make classifications from the presentation of pathological slides. The primary purpose of developing a computer-aided diagnosis system is to assist human pathologists. The implementation of a self-supervised algorithm not only reduces the annotation burden and need for pathological expertise but also, which is more important, increases the data availability of future AI studies. The self-supervised design makes previously useless unlabeled data useful in the pretraining stage. It has the potential to be used in the broadening of disease types (e.g., basal cell carcinoma, squamous cell carcinoma, etc.) and task types (e.g., semantic segmentation of tumor areas in pathologic images based on SSL). From the technical aspect, the combination and comprehensive analysis of multimodal data (H&E staining, immunohistochemical staining, and non-image data like omics data) will be the future research focus.

### Limitations

This study still had several limitations. First, the performance of the algorithm needed improvement as there is still a gap when compared to the state-of-the-art fully supervised learning algorithms. However, to the best of our knowledge, there has been no previous implied SSL algorithm to PCam as a benchmark. When SSL-linear was compared to closed-source fully supervised learning on a public data set, the performance gap was greatly affected by the disparity in training set size. In addition, to prove that SSL-linear achieves better performance than the traditional CNN, more groups with different proportions of pretraining and training sets could be organized both in the ZJU-2 data set and PCam. Second, this study did not include external validation, partly due to the lack of related case slides and difficulties in acquiring such pathological images from external. In the future, the diagnostic ability would be tested on data sets from independent sources (with different races, ages, etc.) to prove the generalization ability.

Additionally, the performance difference of the algorithm could be further investigated based on the above-mentioned different groups, not just malignant or non-malignant groups. Third, this study’s total sample size of eyelid MM was relatively small compared with deep learning studies of other image types. This is limited by the inherent low incidence of eyelid MM. Despite this, all pathological slides are at gigapixel size with large information density and are different from each other; in other words, a total of 524,307 patches as input is relatively sufficient for SSL-linear to achieve considerable performance. Therefore, our sample size is acceptable for a pathological study. Finally, our algorithm can only make a binary classification in this study. In the future, more disease types, including basal cell carcinoma or squamous cell carcinoma, will be introduced to validate the expendability.

In conclusion, SSL-linear was generated and demonstrated considerable performance with higher accuracy than traditional CNNs in distinguishing between benign and malignant eyelid lesions. With less labeled input data and an SSL framework, developing such a diagnostic system is relatively labor-saving and cost-efficient. The implementation of refined algorithms could be further applied to help diagnose various ophthalmic and non-ophthalmic malignancies.

## Data availability statement

The original contributions presented in this study are included in the article/supplementary material, further inquiries can be directed to the corresponding authors.

## Ethics statement

This study was approved by The Second Affiliated Hospital, Zhejiang University School of Medicine (ZJU-2) Ethics Committee (No. Y2019-195) and the study adhered to the Declaration of Helsinki. Written informed consent for participation was not required for this study in accordance with the national legislation and the institutional requirements.

## Author contributions

LW, ZJ, and JY were responsible for study design. LW, AS, and ZL were responsible for data collection and annotation. LW, ZJ, YW, ReG, and RuG analyzed the data. LW, ZJ, GJ, and JY drafted the manuscript. All authors contributed to the article and approved the submitted version.
